# Supplementary material for: Carbapenem-Resistant NDM and OXA-48-like Producing K. pneumoniae: From Menacing Superbug to a Mundane Bacteria; A Retrospective Study in a Romanian Tertiary Hospital
Source: Antibiotics (Basel). 2024 May 12;13(5):435. doi: 10.3390/antibiotics13050435 (PMC11117283; doi:10.3390/antibiotics13050435)
Supplement: Supplementary file 1 [file antibiotics-13-00435-s001.zip › antibiotics-2930828-supplementary.pdf]

Supplementary material

Figure S1. Resistance pattern (%)

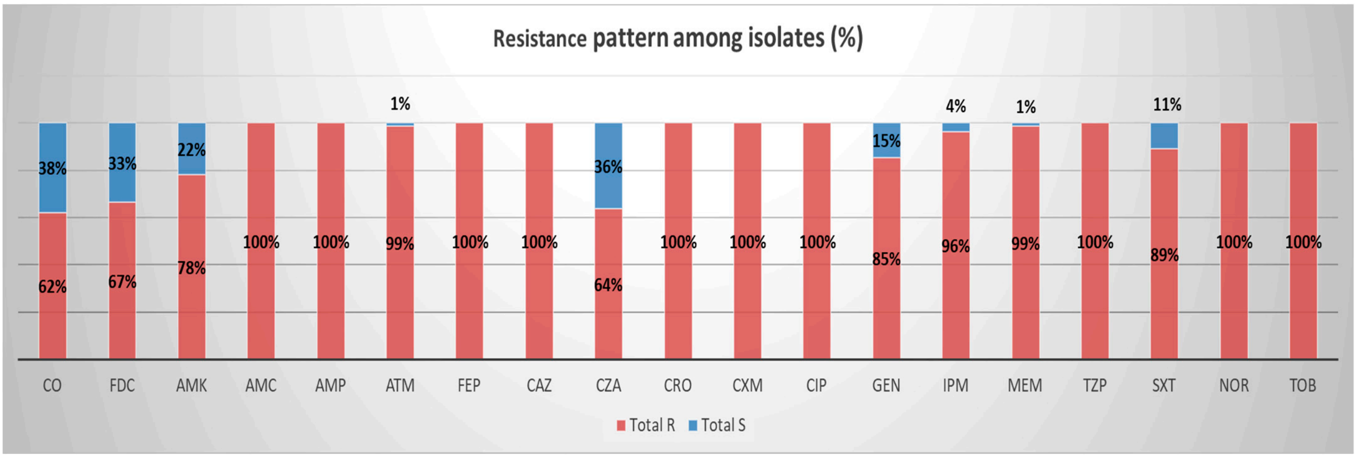

Figure S2. Resistance pattern only for NDM+OXA-48-*like*(%)

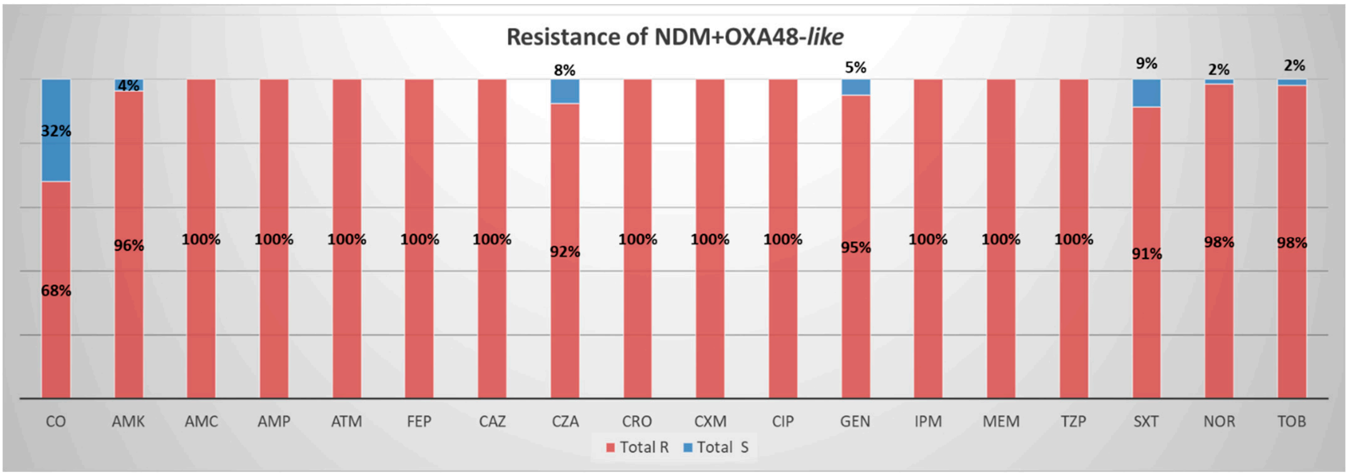

Abbreviations:

Total S= total sensibilities, Total R= total resistance

CO - Colistin
FDC - Cefiderocol
AMK - Amikacin
AMC - Amoxicillin - Clavulanic Acid
AMP - Ampicililin
ATM - Aztreonam
TOB - Tobramycin

FEP - Cefepime
CAZ - Ceftazidime
CZA - Ceftazidime - Avibactam
CRO - Ceftriaxone
CXM - Cefuroxime Axetil
CIP - Ciprofloxacin

GEN - Gentamicin
IPM - Imipenem
MEM - Meropenem
TZP - Piperacillin - tazobactam
SXT - Trimethoprim - Sulfamethoxazole
NOR - Norfloxacin

Table S3. Past hospitalization and distribution of cabapenemases

Carbapenemase type		OXA-48	NDM	KPC	NDM+OXA-48-*like*	Total
		**n=34**	**n=16**	**n=23**	**n=48**	**n=162**
**Previous hospitalization**	**No, n(%)**	3(8.8)	3(18.8)	9(28.1)	16(20.0)	**31(19.1)**
	**Yes, n(%)**	31(91.2)	13(81.3)	23(71.9)	64(80.0)	**131(80.9)**

Table S4. **Comparison of specialties and carbapenemases distribution**

Carbapenemase type		OXA-48	NDM	KPC	NDM+OXA-48-*like*	Total
		**n=31**	**n=13**	**n=23**	**n=64**	**n=131**
**Admission in different medical specialties**	**Surgery wards, n(%)**	10(32.3)	6(46.2)	6(26.1)	18(28.1)	**40(30.5)**
	**Medical wards, n(%)**	21(67.7)	7(53.8)	17(73.9)	46(71.9)	**91(69.5)**

Table S5. **Carbapenem regimen**

**Carbapenem based regimen**	**All n=75(%)**	**Recovered n=32 (%)**	**Deceased n=18 (%)**	**NDM+ OXA-48-*like* n=46 (%)**	**Recovered NDM+ OXA-48-*like* n=22 (%)**	**Deceased NDM +OXA-48-*like* n=12 (%)**
Monotherapy (meropenem)	17(22.3)	5(15.6)	3(16.7)	7(15.2)	4(18.1)	2(16.6)
Combinations of antibiotics	58(77.3)	27(84.38)	15(83.3)	39(84.7)	18(81.8)	10(83.3)
**Carbapenem spared regimen**	**n=76(%)**	**n=30(%)**	**n=25(%)**	**n=31(%)**	**n=11(%)**	**n=12(%)**
Monotherapies	45(59.2)	17(56.6)	16(64.0)	11(35.4)	2(18.1)	5(41.6)
Combinations of antibiotics	31(40.8)	13(43.3)	9(36.0)	20(64.5)	9(81.8)	7(58.3)

Table S6. Colistin based regimen

**Colistin based regimen (n=76)**	**All n=7 (%)**	**Cured n=27 (%)**	**Deceased n=32 (%)**	**NDM+ OXA-48-*like* n=45 (%)**	**Cured NDM+ OXA-48-*like* n=18 (%)**	**Deceased NDM +OXA-48-*like* n=16 (%)**
Monotherapy	15(19.0)	4(14.8)	8(25.0)	6(13.0)	1(5.0)	2(12.5)
Combinations of antibiotics	16(80.0)	23(85.0)	24(75)	39(86.6)	17(95.0)	14(87.5)
**Colistin spared regimen (n=75)**	**n=77(%)**	**n=34(%)**	**n=16(%)**	**n=32(%)**	**n=14(%)**	**n=23(%)**
Monotherapy	47(61.0)	19(55.0)	12(75.0)	12(37.5)	5(35.7)	5(21.7)
Combinations of antibiotics	30(38.0)	15(44.1)	4(25.0)	20(62.5)	9(64.2)	4(17.3)

Table S7. Bivariate analysis of factors associated with patient's outcomes

**variable**	**Survivor n=108**	**Not-survivor n=48**	**RR (%CI)**	**p-value**
**Age, average (S.D.)**	66.19(15.73)	70.96(13.18)	-	0.069
**Gender**				0.058
F	41(63.1)	24(36.9)	-	
M	67(73.6)	24(26.4)	-	
**Disease Severity**				***0.014***
mild	15(93.8)	1(6.3)	reference	-
moderate	76(70.4)	32(29.6)	4.74 (0.69-32.33)	0.112
severe/critical	17(53.1)	15(46.9)	**7.50 (1.08-51.84)**	***0.0411***
**ICU_admission**				***<0.0001***
no	95(79.4)	26(20.6)	reference	-
yes	13(34.2)	22(65.8)	2.92 (1.91-4.47)	***<0.0001***
**Sample type**				***0.001***
other	37(55.2)	30(44.8)	reference	-
urinary	71(79.8)	18(20.2)	0.45(0.28-0.72)	***0.0011***
**Carbapenemase_type**				0.991
OXA-48	21(70.0)	9(30.0)	-	
NDM	10(66.7)	5(33.3)	-	
KPC	22(71.0)	9(29.0)	-	
NDM+OXA-48-*like*	55(68.8)	25(31.3)	-	
**G-neg coinfection**				***0.019***

no	69(76.7)	21(23.3)	reference	-
yes	39(59.1)	27(40.9)	1.75(1.09-2.81)	***0.020***
**G-neg carriage**				***0.026***
no	73(72.3)	28(27.7)	reference	
yes	35(63.6)	20(36.4)	1.31(0.81-2.09)	0.258
**G-neg bacteria coinfection and/or carriage**				0.075
Pseudomonas spp.	10(62.5)	6(37.5)	-	
E.coli	9(75.0)	3(25.0)	-	
Acinetobacter spp.	6(60.0)	4(40.0)	-	
Proteus spp.	3(50.0)	3(50.0)	-	
Providencia spp.	5(83.3)	1(16.7)	-	
**Comorbid neoplasm**				0.661
no	75(68.2)	35(31.8)	-	
yes	33(71.7)	13(28.3)	-	
**Comorbid (diabetes mellitus)**				***0.007***
no	80(76.2)	25(23.8)	reference	
yes	28(54.9)	23(45.1)	1.89(1.19-2.99)	**0.006**
**Transit_urology ward**				***0.033***
no	94(66.7)	47(33.3)	reference	
yes	14(93.3)	1(6.7)	0.10(0.01-0.66)	**0.0175**
**Transit surgery or medical specialties**				0.305
Medical wards	29(74.4)	10(25.6)	-	
Surgery wards	56(65.1)	30(34.9)	-	
**Hospitalisation in the past**				0.480
no	23(74.2)	8(25.8)	-	
yes	90(67.7)	43(32.3)	-	
**UTI/non UTI***				***0.002***
Non UTI	35(55.6)	28(44.4)	reference	
UTI	73(78.5)	20(21.5)	0.48(0.30-0.77)	***0.0014***
**Carbapenem based regimen**				0.058
Carbapenem spared	47(62.7)	28(37.3)	reference	
Carbapenem based	54(77.1)	16(22.9)	0.61(0.36-1.03)	0.0648
**Ceft-Avi based regimen**				0.198
Ceft-Avi spared	81(72.3)	31(27.7)	-	
Ceft-Avi based	20(60.6)	13(39.4)	-	
**Colistin based regimen**				***0.008***
Colistin -spared	60(77.6)	17(22.1)	reference	
Colistin -based	44(57.9)	32(42.1)	1.90(1.16-3.13)	

*UTI : urinary tract infection
